# Endoscopic endonasal versus open approach for craniopharyngioma treatment: a systematic review of clinical characteristics

**DOI:** 10.1007/s00381-025-06788-3

**Published:** 2025-03-17

**Authors:** Geena Jung, Joshua M. Cohen, David Oriko, Emery Buckner-Wolfson, Timothy Kim, Genesis Liriano, Andrew J. Kobets

**Affiliations:** 1 Montefiore Medical Center, Albert Einstein College of Medicine, Bronx, NY USA; 2 University of Maryland Medical Center, Baltimore, MD USA; 3 Department of Neurosurgery, Montefiore Medical Center, Bronx, NY USA

**Keywords:** Craniopharyngioma, Endoscopic endonasal, Transcranial, Suprasellar

## Abstract

**Background:**

Craniopharyngiomas are rare tumors found in the suprasellar region of the brain. Untreated, they have the potential to cause debilitating complications, including vision loss and cognitive decline. Craniopharyngiomas can be resected through several surgical options including endoscopic endonasal (EEA) and open, transcranial approaches, cystic drainage, and ventricular neuroendoscopic approaches. Here, we seek to review the literature and compare characteristics of lesions resected by the EEA versus open approach.

**Methods:**

A comprehensive database search was performed on PubMed, Google Scholar, and Embase using key terms. Included studies utilized both EEA and open approaches for craniopharyngioma resection.

**Results:**

No studies reported a significant difference in tumor location, consistency, pathology, or presence of calcification. One study reported an increased preoperative tumor volume with the open approach. The open approach was significantly associated with a longer follow-up period (4/16) and hospital length of stay (2/16), as well as a greater rate of recurrence (2/16) and mortality (1/16). New-onset diabetes insipidus (3/16) and vision deterioration (3/16) following surgery were significantly more common following an open approach.

**Conclusion:**

Inherent in the surgical decision-making regarding approach are the anatomical considerations of the tumor. Through our literature search, we found tumors were not substantially different for the different approaches, consistent with our clinical experience. This may be related to the refinement of endonasal techniques, allowing larger, suprasellar tumors to be amenable to GTR more than in the past.

## Introduction

Craniopharyngiomas (CPs) are rare, benign intracranial tumors that typically arise in the suprasellar region of the brain. They are found near the pituitary gland and frequently involve structures including the hypothalamus, third ventricle, anterior cerebral artery complex, optic chiasm, and cranial nerves [1–3]. Histologically, CPs can be characterized into one of two primary subtypes: papillary and adamantinomatous, with the latter being most common [4–6]. Left untreated, CPs can lead to significant neurologic complications including vision loss and pituitary insufficiency [5–9].

Despite their consideration as a benign lesion, the intricate location of craniopharyngiomas and diverse clinical manifestations make surgical treatment a challenge for physicians [3]. The proximity to neuroendocrine tissues of the pituitary, the pituitary stalk, and hypothalamic structures create potential treatment problems [10, 11]. CPs can be treated via microsurgical tumor resections or minimally invasive alternatives such as stereostatic procedures. These alternative procedures can treat tumors with cystic components and include shunt catheters for permanent cystic drainage, cystic-ventricular endoscopic approaches for “self-washing out,” interferon injections, and BRAF/MEK inhibitors. [12–15].

The two most common approaches for craniopharyngioma resection include an endoscopic endonasal (EEA) and transcranial (open) approach [6]. Transcranial approaches require an extent of brain retraction and greater manipulation of the adjacent neurovascular structures [1]. An endoscopic endonasal approach allows advancement of a light source and lens, increasing the field of view [9]. While both approaches are used for craniopharyngioma resection, the indications for each have not been clearly delineated [16–23].

Here, we seek to review the literature and compare the tumor and imaging characteristics of craniopharyngiomas resected by the EEA versus the open approach. Our goal is to better understand the indications for each surgical approach. Our secondary aims are to evaluate differences in the clinical course and postoperative complications between the two patient groups.

## Methods

A systematic search study using PubMed, Embase, and Google Scholar was conducted from database inception to May 2024. The following search criteria was used: “craniopharyngioma” AND (“endoscopic” OR “craniotomy”). Studies were uploaded into Covidence (Cochrane, London, UK) and screened based on inclusion and exclusion criteria. Inclusion criteria involved the following: papers comparing endonasal endoscopic (EEA) and open approaches for craniopharyngioma resection. Studies were not included if they only utilized one approach (e.g., an institutional study looking at the outcomes of EEA approaches for craniopharyngiomas were excluded). Studies were also excluded if they were case reports, systematic reviews, meta-analyses, or not written in English. Prior reviews were not included, given the likely overlap in included studies. Case reports were also not included, as we specifically were looking for papers that directly compared endoscopic versus open, transcranial approaches for craniopharyngioma resection with multiple patients. Of note, given the limited number of studies focusing on specific age groups, we included papers that involved pediatric patients, adult patients, or both.

**Fig. 1:**
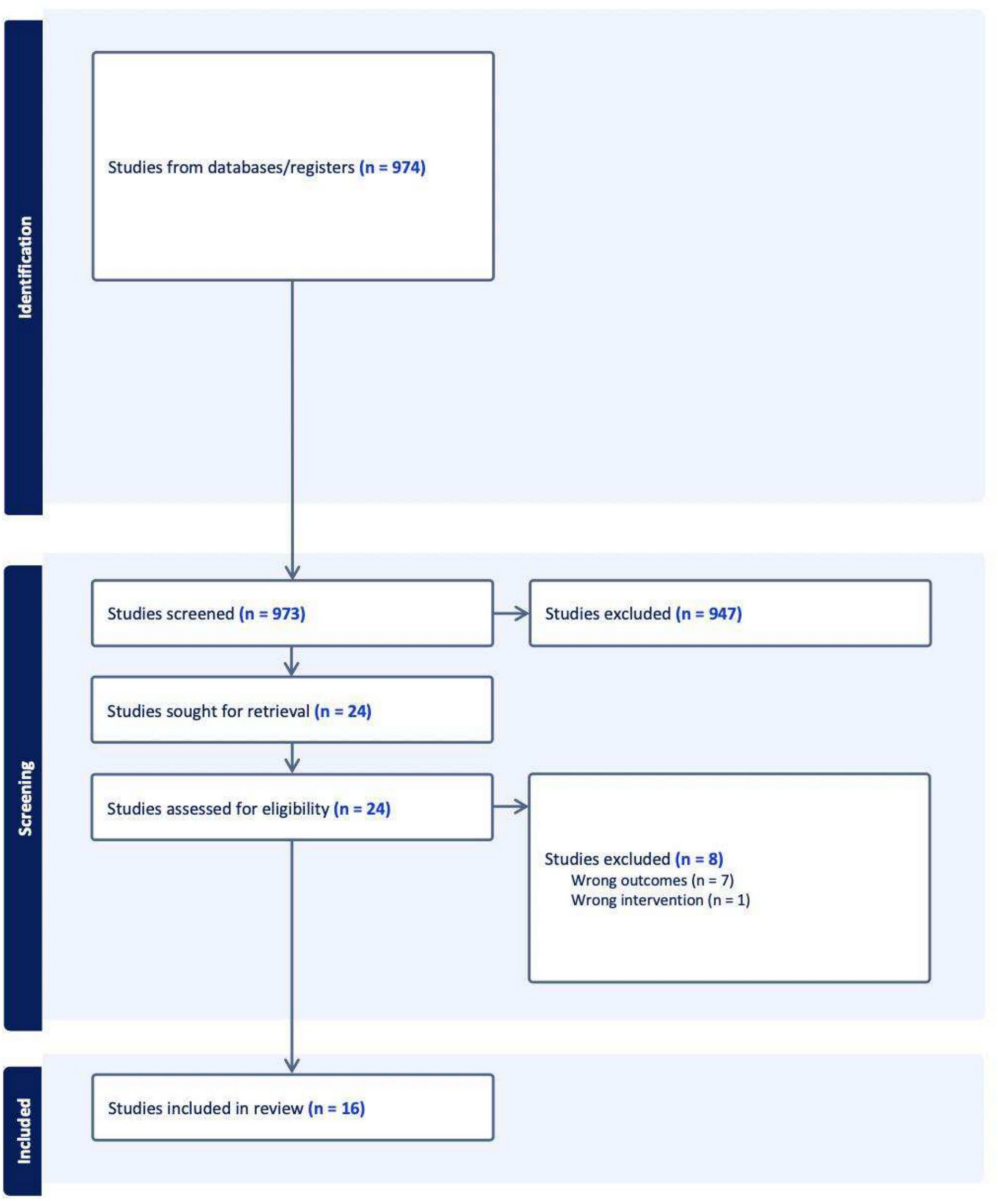
PRISMA flow diagram

After screening for eligible studies, abstract screening, full-text review, and data extraction were performed. In accordance with the PRISMA 2020 guidelines, a rigorous assessment of the quality of each study was performed to ensure the evidence was reliable and methodologically sound. This quality assessment involved evaluating each paper’s study design, risk of bias, sample size, data collection methods, and statistical analysis to determine its suitability for inclusion in this review. Only studies meeting predefined criteria for methodological rigor were included to ensure the quality of the overall findings. Two authors (GJ and JC) independently reviewed titles, abstracts, and full texts using Covidence (Fig. 1). Any discrepancies in data extraction were resolved with discussions with the senior author (AJK).

## Results

After removal of duplicate studies, a total of 973 studies were obtained from the initial search criteria. A total of 16 studies met inclusion criteria and were deemed suitable for data extraction and analysis. All but one of the included studies were retrospective in nature. In all of these studies, both transcranial and endoscopic approaches were available during the study period, and patients underwent surgery using one of these techniques. However, none of the studies provided specific indications or criteria for selecting one approach over the other in their methodology sections.

We first looked at demographic characteristics of patients included in the studies. Among the 15 studies that reported mean age at presentation, only one study found a significant difference between the EEA and open group (48.5 years versus 31.2 years, respectively, *p* = 0.04) [30] (Table 1). Among the 15 studies that reported sex of patients, two studies reported significant differences between the two groups. Govindarajan et al. found that the open group consisted of 52.4% males, while the EEA group consisted of 44.2% males (*p* = 0.0001) [24]. Parasher et al. reported that the EEA group was 83.3% males, while the open group was 30.0% males (*p* = 0.027) [34]. Four studies reported patient race, and 2 (50%) found significant differences between the two groups [22, 23]. In both studies, white patients made up the majority.

**Table 1:** Demographic characteristics of patient population

Author	Indications for study	Number of patients			Mean age (years)			Sex (male)			Race/ethnicity				
		EEA	Open	Total	EEA	Open	*p* value	EEA	Open	*p* value	EEA		Open	*p* value	
Abiri et al. (2022) [22]	Patients diagnosed with craniopharyngioma between 2010 and 2017 who underwent surgery	508	1213	1714	43.1 ± 20.1	41.3 ± 21.9	0.189	48.8%	49.2%	0.852	Black: 17.0% White: 76.8% Asian: 6.2%		Black: 21.2% White: 74.5 Asian: 4.2%	**0.049**	
Fan et al. (2021) [23]	Patients diagnosed with craniopharyngiomas and surgically treated between 2006 and 2016	125	190	315	42.5 ± 15.8	40.9 ± 16.2	nr	57.6%	58.9%	0.79	N/A				
Govindarajan et al. (2021) [24]	Patients over the age of 18 with a diagnosis of craniopharyngiomas who were surgically treated between 1998 to 2014	4655	6511	11166	44.77 (median)	48.43 (median)	nr	44.2%	52.4%	**0.0001**	Black: 15.2% White: 61.10% Asian: 7.04%		Black: 16.25% White: 62.29% Asian: 3.11%	0.0049*	
Jeswani et al. (2016) [25]	Patients with craniopharnygiomas and similar midline suprasellar tumors surgically treated between 2000 and 2013	19	34	53	45 ± 17.0	45.3 ± 19.8	0.96	47.4%	44.1%	0.55	N/A				
Lehrich et al. (2021) [26]	Patients diagnosed with craniopharyngiomas from 2004–2015	70	267	337	N/A			N/A			N/A				
Li et al. (2019) [27]	Patients with craniopharyngiomas who were treated between 2011 and 2015	58	59	117	43.59 ± 14.98	40.31 ± 15.52	nr	47.1%	57.7%	0.496	N/A				
Li et al. (2023) [28]	Patients over the age of 18 with a diagnosis of craniopharyngioma who were treated between 2018 and 2022	58	59	117	42.84 ± 12.74	41.02 ± 10.2	0.393	36.2%	37.3%	0.904	N/A				
Madsen et al. (2019) [28]	Patients under the age of 18 who were treated between 2001 to 2017	28	15	43	8.9	6.6	nr	73.50%	41.10%	nr	N/A				
Marx et al. (2021) [30]	Patients with a suprasellar craniopharyngioma who were treated between 2001 and 2018	17	13	30	48.5	31.2	**0.04**	53%	38%	0.48	N/A				
Moussazadeh et al. (2016) [31]	Patients aged 18 years and older with craniopharyngiomas who were treated between 2000 and 2015	21	5	26	50.9 ± 13.4	50.0 ± 25.2	0.92	23.8%	40%	0.48	N/A				
Nie et al. (2022) [32]	Patients who underwent cranopharyngioma resection between 2010 and 2019	88	185	273	37.8 ± 13.8	38.2 ± 12.3	0.37	46.6	47.0%	1	N/A				
Ozgural et al. (2018) [33]	Patients with craniopharyngiomas who underwent surgery between 2013 and 2017 at an institution	11	13	24	37.4	51	Ns	54.5%	60.2%	ns	N/A				
Parasher et al. (2020) [34]	Pediatric patients undergoing craniopharyngioma resection between 2003 and 2014	12	10	22	7.92	6.4	0.297	83.3%	30.0%	**0.027**	White: 75%	White: 70%			> 0.999
Parasher et al. (2022) [35]	Patients with craniopharyngiomas undergoing treatment from 2001 to 2017	14	22	36	46.3	44.8	0.793	57.1%	54.5%	0.733	Black: 21.4% White: 78.6% Other: 0.0%	Black: 36.4% White: 54.5% Other: 9.1%			0.263
Wannemuehler et al. (2016) [36]	Adult patients (18 years and older) undergoing primary resection of a craniopharyngioma between 2005 and 2015	9	12	21	52.4	48.3	0.59	66.7%	58.3%	1	N/A				
Wu et al. (2022) [37]	Pediatric patients with craniopharyngiomas (< 18 years at diagnosis) who had surgery between 2009 and 2021	35	16	51	12 (median)	11.5 (median)	0.737	62.9%	43.8%	0.201	N/A				

We next looked at symptoms at presentation between those operated with a an endoscopic versus open, transcranial approach (Table 2). Three studies were excluded due to lack of information regarding preoperative symptoms [22, 24, 26]. Of the 11 studies that reported visual impairment, 7 studies that reported headache, 5 studies that reported diabetes insipidus, and 3 studies that reported hypopituitarism, none found any significant differences between the EEA and open groups. Five studies reported hydrocephalus as a symptom at presentation, and two of these studies found that hydrocephalus was more common in the open group (75% versus 32.1%, *p* = 0.01 and 86% versus 25%, *p* = 0.02) [29, 34]. Finally, five studies reported on hypogonadism, and one of these studies found that hypogonadism was more common in the endoscopic group (60.0% versus 18.8%, *p* = 0.006) [37].

**Table 2:** Preoperative symptoms of patients with craniopharyngiomas resected by EEA versus open approach

Author	Visual impairment			Headache			Hydrocephalus				Diabetes insipidus			Hypopituitarism			Hypogonadism		
	EEA	Open	*p* value	EEA	Open	*p* value	EEA	Open		*p* value	EEA	Open	*p* value	EEA	Open	*p* value	EEA	Open	*p* value
Fan et al. (2021) [19]	67.2%	65.3%	0.92	49.6%	52.6%	0.57	N/A				17.6%	16.3%	0.61	35.2%	33.2%	0.68	N/A		
Jeswani et al. (2016) [21]	57.9%	52.9%	0.73	26.3%	26.5%	0.99	N/A				N/A			N/A			15.8%	2.9%	nr
Li et al. (2019) [23]	88.2%	76.9%	0.595	23.5%	42.3%	0.207	N/A				29.4%	30.8%	0.925	N/A			56.3%	75.0%	0.215
Li et al. (2023) [24]	N/A			N/A			N/A				N/A			12.1%	10.2%	0.744	31.0%	18.6%	0.121
Madsen et al. (2019) [25]	14.3%	20%	0.63	N/A			32.1%	75%		**0.01**	N/A			N/A			N/A		
Marx et al. (2021) [26]	Visual acuity impairment: 39% Visual field impairment: 54%	Visual acuity impairment: 42% Visual field impairment: 62%	ns	N/A			N/A				24%	15%	ns	N/A			41%	38%	ns
Moussazadeh et al. (2016) [27]	10/21 (47.6)	3/5 (60)	0.64	19.0%	20%	0.96	N/A				N/A			N/A			N/A		
Nie et al. (2022) [28]	53.4%	47.0%	0.37	80.7%	70.8%	0.1	12.5%	15.1%	0.71		N/A			N/A			N/A		
Ozgural et al. (2018) [29]	81.8%	76.9%	nr	N/A			9.1%	53.8%	nr		N/A			N/A			N/A		
Parasher et al. (2020) [30]	N/A			N/A			25%	86%	**0.02**		N/A			N/A			N/A		
Parasher et al. (2022) [31]	78.6%	68.2%	0.706	N/A			50%	27.3%	0.286		N/A			N/A			N/A		
Wannemuehler et al. (2016) [32]	88.9%	83.3%	1	66.7%	41.7%	0.39	N/A				0.0%	0.0%	nr	33.3%	33.3%	1	N/A		
Wu et al. (2022) [33]	42.9%	56.3%	0.374	68.6%	50.0%	0.203	N/A				31.4%	25.0%	0.892	N/A			60.0%	18.8	**0.006**

Next, data related to tumor and imaging characteristics were extracted (Table 3). No significant differences in tumor location (e.g., sellar versus suprasellar), consistency (e.g., solid versus mixed versus cystic), pathologic type (e.g., adamantinomous versus papillary), or presence of calcification were observed. Abiri et al. found that a larger preoperative tumor volume was present in the open group (*p* < 0.001) [22].

**Table 3:** Preoperative tumor characteristics

Author	Tumor Volume (cm3)			Tumor Location					Tumor Consistency				Pathologic Type		Calcification		
	EEA	Open	*p* value	EEA	Open		*p* value		EEA	Open	*p* value	EEA	Open	*p* value	EEA	Open	*p* value
Abiri et al. (2022) [22]	≤ 3 cm: 70.2% > 3 cm: 29.8%	≤ 3 cm: 56.7% > 3 cm: 43.3%	< 0.01*	N/A					N/A			Adamantinomas: 38.4% Papillary: 13.0% NOS: 48.6%	Adamantinomas: 44.0% Papillary: 12.0% (NOS): 43.9%	0.106	N/A		
Fan et al. (2021) [19]	26.6 ± 9.1	33.3 ± 12.8	0.35	N/A					Solid: 11.2% Mixed: 66.4% Cystic: 22.4%	Solid: 10.5% Mixed: 67.4% Cystic: 22.1%	Solid: 0.87 Mixed: 0.93 Cystic: > 0.99	Adamantimous: 78.4% Papillary: 21.6%	Adamantimous: 73.7% Papillary: 26.3%	Adamantimous: 0.89 Papillary: 0.57	N/A		
Jeswani et al. (2016) [21]	7.8 ± 6.5	10.5 ± 12.5	0.12	N/A					Solid: 26.3% Mixed: 57.9% Cystic: 15.8%	Solid: 23.5% Mixed: 47.1% Cystic: 26.5%	Solid: 0.5 Mixed: 0.57 Cystic: 0.5	Adamantimous: 68.8% Papillary: 31.3%	Adamantimous: 69.7% Papillary: 27.3%	Adamantimous: 1 Papillary: 1	N/A		
Li et al. (2019)23				N/A					Solid: 17.6% Mixed: 52.9% Cystic: 29.4%	Solid: 30.8% Mixed: 61.5% Cystic: 3.8%	0.072	N/A			58.8%	61.5%	1
Li et al. (2023) [24]	9.89	9.22	ns	N/A					N/A			Adamantimous: 67.2%	Adamantimous: 55.9%	0.209	65.5%	66.1%	0.947
Madsen et al. (2019) [25]	6.244 (median)	18.429 (median)	0.06	Sellar: 1/28 (3.6) Sellar-suprasellar: 12/28 (42.9) Suprasellar: 15/28 (53.6)		Sellar: 0/15 (0.0) Sellar-suprasellar: 6/15 (40.0) Suprasellar: 9/15 (60.0)		Sellar: 0.46 Sellar-suprasellar: 0.86 Suprasellar: 0.69	N/A	N/A	N/A	N/A	N/A	N/A	N/A	N/A	N/A
Moussazadeh et al. (2016) [27]	8.5 ± 5.9	13.9 ± 7.8	0.1	Sellar-suprasellar: 19.0% Suprasellar: 57.1%		Sellar-suprasellar: 20% Suprasellar: 60%		Nr	N/A			Adamantinomas: 33.3% Papillary: 14.3% NOS: 52.4%	Adamantinomas: 60% Papillary: 0% NOS: 40%	Adamantinomas: 0.29 Papillary: 0.39	66.7%	3.3%	0.31
Nie et al. (2022) [28]	8.2 ± 7.9	8.7 ± 7.1	0.48	N/A					Solid: 20.5% Mixed: 53.4% Cystic: 26.1%	Solid: 22.1% Mixed: 47.1% Cystic: 30.8%	Solid: 0.88 Mixed: 9.37 Cystic: 0.48	Adamantinoma: 87.5% Papillary: 12.5%	Adamantinomas: 91.9% Papillary: 8.1%	Adamantinomas: 0.27 Papillary: 0.27	N/A		
Ozgural et al. (2018) [29]	24.575	37.897	nr	N/A					N/A			N/A			N/A		
Parasher et al. (2020) [30]	N/A	N/A	N/A	Cavernous sinus extension: 8.3% Suprasellar extension: 91.7%		Cavernous sinus extension: 13% Suprasellar extension: 100%		> 0.999	N/A			N/A			N/A		
Parasher et al. (2022) [31]	6.998	8.560	0.603	Sellar: 0.0% Suprasellar: 85.7% Intraventricular: 14.3%		Sellar: 9.1% Suprasellar: 77.3% Intraventricular:3.6%		0.509	Solid: 14.3% Mixed: 50.0% Cystic: 35.7%	Solid: 27.3% Mixed: 40.9% Cystic: 31.8%	0.655	N/A			N/A		
Wannemuehler et al. (2016) [32]	4.6 ± 4.7	7.8 ± 5.0	0.16	N/A					Solid: 0.0% Mixed: 66.7% Cystic: 33.3%	Solid: 0.0% Mixed: 66.7% Cystic: 33.3%	Solid: 1 Mixed: 1 Cystic: 1	Adamantinoma: 66.7% Papillary: 22.2 Mixed: 11.1%	Adamantinomas: 91.7% Papillary: 8.3% Mixed: 0.0%	Adamantinomas: 0.27 Papillary: 0.55 Mixed: 0.43	N/A		
Wu et al. (2022) [33]	14.6 (median)	16.7 (median)	0.543	Sellar: 3/35 (8.6) Sellar-suprasellar: 16/35 (45.7) Suprasellar: 16/35 (45.7)		Sellar: 0/16 (0.0) Sellar-suprasellar: 6/16 (37.5) Suprasellar: 10/16 (62.5)		0.501	Solid: 14.3% Mixed: 57.1% Cystic: 28.6%	Solid: 6.3% Mixed: 50.0% Cystic: 43.8%	0.572	N/A			68.6%	81.3%	0.546

Significant differences in extent of resection between the EEA and open groups were observed (Table 4). Three out of 13 studies found that GTR was significantly more common in the EEA group. Rates of GTR ranged from 13.4 to 94.3% in the endoscopic group and 12.8 to 90.5% in the open group [22, 23, 37]. Three out of seven studies reported that adjuvant radiotherapy was significantly more common following open surgery as compared to endoscopic surgery. Moussazadeh et al. reported that 60% of patients at an institution required radiotherapy following open surgery [31]. Four out of 10 studies found that the follow-up period was significantly less in the EEA group, and 2/6 found that the average hospital length of stay was significantly less in the EEA group. One study found that the open group was associated with a greater rate of mortality (2.13% versus 0.27%, *p* < 0.0001) [24]. No significant differences in rates of recurrence were observed in any of the studies.

**Table 4:** Hospital course

Author	Extent of resection			Adjuvant radiotherapy			Average length of stay (days)			Follow-up period (months)			Mortality			Recurrence		
	EEA	Open	*p* value	EEA	Open	*p* value	EEA	Open	*p* value	EEA	Open	*p* value	EEA	Open	*p* value	EEA	Open	*p* value
Abiri et al. (2022) [18]	GTR: 13.4% STR: 86.6%	GTR: 12.8% STR: 82.7%	0.732	15.2%	22.3%	** < 0.001**	8 ± 11.4	10.5 ± 12.2	< 0.001*	N/A			N/A			N/A		
Fan et al. (2021) [19]	GTR: 91.2% STR: 1.6% NTR: 7.2%	GTR: 90.5% STR: 1.6% NTR: 7.9%	GTR: 0.85 STR: > 0.99 NTR: 0.95	N/A			N/A			2.78	2.96	0.53	2.4%	2.6%	0.72	6.4%	8.9%	0.35
Govindarajan et al. (2021) [20]	N/A			N/A			N/A			N/A			0.27%	2.13%	** < 0.0001**	N/A		
Jeswani et al. (2016) [21]	N/A			N/A			N/A			33.58	34.46	0.8	5.3%	2.9%	1	N/A		
Lehrich et al. (2021) [22]	GTR: 26.5% STR: 73.5%	GTR: 20.8% STR: 79.1%	0.47	10.0%	26.6%	**0.003**	9.3 ± 16.7	10.6 ± 9.3	0.54	N/A			30 day mortality: 0.0% 90 day mortality: 1.4%	30 day mortality: 1.5% 90 day mortality: 1.9%	30 day: 0.30 90 day: 0.80	N/A		
Li et al. (2019) [23]	GTR: 64.7% STR: 35.3%	GTR: 65.4% STR: 34.6%	0.964	N/A			N/A			5.5	9	0.052	N/A			N/A		
Li et al. (2023) [24]	GTR: 91.4% STR: 8.6%	GTR: 78.0% STR: 22.0%	0.027*	N/A			N/A			25.5	26	0.724	N/A			N/A		
Madsen et al. (2019) [25]	GTR: 85.7% STR: 14.3%	GTR: 53.3% STR: 46.7%	**GTR: 0.03** **STR: 0.05**	10.7%	20.0%	0.71	13 (median)	15.5 (median)	0.17	14	83	** < 0.001**	3.6%	0.0%	0.75	N/A		
Marx et al. (2021) [26]	GTR: 59% STR: 18% Partial: 23%	GTR: 54% STR: 38% Partial: 8%	ns	35.3%	15%	ns	N/A			56	136	**0.002**	0.0%	8%	ns	N/A		
Moussazadeh et al. (2016) [27]	GTR: 90.5% NTR: 9.5%	GTR: 40% NTR: 60%	0.009*	9.5%	60%	0.002*	9.3 ± 6.6	15.0 ± 7.9	0.11	30.1 ± 28.9	56.8 ± 54.1	0.13	N/A			N/A		
Nie et al. (2022) [28]	N/A			N/A			N/A			N/A			0.0%	0.0%	1	N/A		
Ozgural et al. (2018) [29]	GTR: 81.8% STR: 18.2%	GTR: 30.8% STR: 69.2%	nr	N/A			N/A			N/A			N/A			N/A		
Parasher et al. (2020) [30]	GTR: 83.3%	GTR: 70.0%	0.624	N/A			N/A			58.86	86.16	**0.006**	N/A			N/A		
Parasher et al. (2022) [31]	GTR: 64.3% STR: 33.3% Partial: 7.1%	GTR: 59.1% STR: 31.8% Partial: 9.1%	0.948	N/A			10.6	21.5	**0.024**	N/A			N/A			N/A		
Wannemuehler et al. (2016) [32]	GTR: 55.5% STR: 44.4%	GTR: 58.3% STR: 41.7%	GTR: 1 STR: 1	22.2%	25.0%	nr	10.1 ± 5.4	14.4 ± 15	0.38	7.1	12.3	0.57	0.0%	0.0%	nr	11.1%	11.1%	1
Wu et al. (2022) [33]	GTR: 94.3% STR: 5.7%	GTR: 75.0% STR: 25.0%	0.13	5.7%	25.0%	0.13	N/A			40.5	66	**0.007**	2.9%	6.3%	0.533	5.9%	13.3%	0.755

Numerous differences in postoperative complications were reported (Table 5). Of the eight studies that reported visual complications, three found that there was a significantly greater proportion of these complications within the open group [17, 23, 24, 37]. Rates reached as high as 38.8% in the open group compared to 10% in the endoscopic group [25, 31]. CSF leak was found to be significantly more common in the EEA group in 4/9 studies, and diabetes insipidus was more common in the open group in 3/9 studies. Moussazadeh et al. found that meningitis was significantly more common following an open approach (20% versus 0%, *p* = 0.04) [31]. No differences in rates of hydrocephalus were found. Two studies reported that postoperative hypopituitarism was significantly more prevalent in the open group [29, 32].

**Table 5:** Postoperative complications

Author	Worsening of visual symptoms			Diabetes insipidus			CSF leak			Meningitis			Hydrocephalus			Hypopituitarism		
	EEA	Open	p value	EEA	Open	p value	EEA	Open	p value	EEA	Open	p value	EEA	Open	p value	EEA	Open	p value
Fan et al. (2021) [19]	1.6%	11.0%	**< 0.001**	50.4%	52.6%	0.55	12.0%	0.5%	< **0.001**	7.2%	4.7%	0.45	9.6%	7.4%	0.65	12.8%	13.7%	0.82
Govindarajan et al. (2021) [20]	2.75%	5.90%	**0.0031**	11.70%	25.14%	** < 0.0001**	1.42%	0.47%	** < 0.0001**	0.41%	0.31%	0.893	1.07%	10.41%	< 0.0001	6.99%	13.76%	0.154
Jeswani et al. (2016) [21]	0.0%	38.8%	nr	31.6%	52.9%	nr	26.3%	0.0%	**0.004**	5.3%	0.0%	nr	15.8%	8.8%	0.69	42.1%	38.2%	nr
Li et al. (2019) [23]	N/A			64.7%	61.5%	0.834	N/A			N/A			N/A			N/A		
Li et al. (2023) [24]	N/A			N/A			N/A			N/A			N/A			45%	58.5%	0.019
Madsen et al. (2019) [25]	N/A			N/A			N/A			N/A			N/A			92.8%	100%	0.76
Marx et al. (2021) [26]	Visual Acuity: 9% Visual Field: 0%	Visual Acuity: 0% Visual Field: 8%	Visual Acuity: ns Visual Field: 0.14	41%	62%	ns	29%	15%	ns	12%	0%	ns	12%	0%	ns	N/A		
Moussazadeh et al. (2016) [27]	10%	0%	0.48	N/A			5%	0/5 (0)	0.63	0%	20%	**0.04**	N/A			N/A		
Nie et al. (2022) [28]	N/A			51.1%	72.4%	** < 0.01**	4.5%	0.0%	**0.01**	2.3%	2.2%	1	N/A			53.4%	68.1%	0.02*
Ozgural et al. (2018) [29]	0/11 (0.0)	0/13 (0.0)	nr	N/A			N/A			N/A			N/A			N/A		
Parasher et al. (2022) [31]	N/A			78.6%	90.9%	0.357	21.4%	0.0%	0.051	N/A			N/A			N/A		
Wannemuehler et al. (2016) [32]	0.0%	25.0%	0.23	55.5%	50.0%	1	22.2%	0.0%	0.17	0.0%	8.3%	1	0.0%	0.0%	nr	33.3%	16.7%	0.61
Wu et al. (2022) [33]	0.0%	25.0%	**0.012**	29.4%	60.0%	**0.043**	5.7%	0.0%	> 0.99	N/A			2.9%	12.5%	0.229	Partial hypopituitarism: 35.3% Panhypopituitarism: 47.1%	Partial hypopituitarism: 20.0% Panhypopituitarism: 80.0%	Partial hypopituitarism: 0.463 Panhypopituitarism: 0.032

## Discussion

Craniopharyngiomas are benign intracranial tumors that typically arise near the pituitary gland and often involve surrounding structures including the hypothalamus, third ventricle, optic chiasm, and cranial nerves. While these tumors may arise anywhere along the craniopharyngeal canal, 95% have a suprasellar component, with 5% being purely intrasellar [38]. Their slow growth leads to a delay in symptom presentations, ranging months to many years. The two most common approaches for craniopharyngioma resection include an endoscopic endonasal and transcranial approach. Here, we review the existing literature regarding tumor and imaging characteristics of lesions resected by the EEA versus open, transcranial approach to better understand indications for each approach. We also evaluate differences in the clinical course between the two groups. Notably, due to the limited number of studies focusing exclusively on pediatric patients, both adult and pediatric cases have been included in the present analysis.

We first looked at demographic characteristics of patients and found that a majority of studies reported no differences between the two approaches. Only one study reported a difference in mean age at presentation, two reported differences in sex of patients, and two reported racial differences. Similarly, symptoms at presentation including visual impairment, headache, and hypopituitarism did not significantly differ between the EEA and open group for a majority of papers.

Inherent in the surgical decision-making regarding approach are the anatomical considerations of the tumor. To better understand why an endoscopic approach would be favored over an open approach (or vice versa), we extracted data regarding differences in tumor size, location, consistency, pathologic subtype, and the presence of calcification. Traditionally, the open approach has been favored for its capability to achieve more extensive surgical resections. As such, we predicted that larger tumors would be more amenable to resection by the open approach, given the freedom of movement and maneuverability the approach offers. Moreover, we hypothesized that tumors located above the sella turcica or extending into the third ventricle would be more likely to be resected by an open approach.

Interestingly, we found that tumor characteristics (e.g., location, consistency, pathologic subtype, presence of calcification) through the literature search were not substantially different for the different approaches. Only one study (Abiri et al.) found a larger preoperative tumor volume was present in the open group. While these findings may reflect surgeon preferences or training influences, they also raise intriguing questions about the evolution and utility of endoscopic surgery. Over the past decade, endoscopic techniques have advanced significantly, and have likely led surgeons to resect tumors that were previously considered suitable only for open surgery. The improvements in endoscopic instruments and 3D and 4 K technology have provided enhanced visualization of tumor margins and surrounding structures, allowing for more extensive and precise resections. These advancements may explain why we found no significant difference in tumor characteristics in the literature between the two approaches.

The advances in endoscopic surgery may also be reflected by its higher rates of GTR. Our review of the literature revealed that the endoscopic group was associated with increased rates of GTR in 3/13 studies. Several authors proposed that there is better visualization of the sellar anatomy with endoscopic approaches, allowing for more confidence in achieving adequate margins. It is important to note, however, that some institutions favor less aggressive surgeries with subsequent adjuvant radiotherapy. This is a particularly important confounding variable that should be accounted for, especially when comparing rates of GTR between different institutional studies.

Finally, we looked at postoperative complications and found that with the exception of CSF leaks, postoperative complications were generally more common in the open group compared to the EEA group. For instance, visual impairment (three out of eight studies), diabetes insipidus (three out of nine studies), and meningitis (one out of seven studies) were found to be significantly more common in the open group. It has been hypothesized that endoscopic surgery results in fewer complications because it is less likely to result in injury or manipulation to surrounding structures such as the optic nerves when compared to open, transcranial approaches. These decreased rates of postoperative complications in the endoscopic group may reflect the better quality of life the approach offers to patients, especially in relation to visual and endocrine function.

It is important to note that our systematic review is not without limitations. There are limitations in the study’s search strategy. It is possible that relevant studies may have been excluded due to omission of terms or phrases from the initial search. In addition, it is not possible to capture all the variabilities in the 16 studies with relation to the tumor characteristics or postoperative complications recorded. Further, the inclusion of both adult and pediatric cases potentially impacts the analysis due to craniopharyngiomas exhibiting distinct characteristics and behaviors in these populations. Further studies focusing on distinct age groups are warranted. The reported outcomes varied between the studies and as such, we were not able to encompass all the data presented in the included studies. Significant variability was observed in terms of patient demographics, tumor characteristics, surgical techniques, and follow-up protocols, which further complicated data integration. In addition, many studies employed different definitions or classification systems for reporting outcomes, such as visual outcomes or meningitis, which limited the standardization of the data for analysis. While we acknowledge that a meta-analysis could have enriched the study, the substantial variability across studies prevented meaningful aggregation of the data. Given these challenges, we decided to report the individual study outcomes to provide a more nuanced comparison between the EEA and open approaches. We believe that future studies with more standardized methodologies will enable more robust analyses and contribute to a deeper understanding of these treatment approaches. Finally, we recognize that while craniopharyngioma resection is typically done via an endoscopic or open approach, there are times as well where the surgical plan is to stage the procedure with one approach first followed by the second.

## Conclusions

Overall, we report no significant differences in tumor characteristics resected by the endoscopic versus open, transcranial approach. While these findings may reflect surgeon preferences or training influences, they also underscore the significant advancements made in endoscopic surgical techniques. Historically, the open approach was favored due its ability to achieve more extensive surgical resections. However, advancements in endoscopic techniques and tools have likely now allowed surgeons to resect tumors once thought to be suitable only for open surgery. These advancements are also evidenced by the higher rates of GTR and lower incidence of postoperative complications observed in the endoscopic group.

## Data Availability

No datasets were generated or analysed during the current study.
